# Accelerated sparsity based reconstruction of compressively sensed multichannel EEG signals

**DOI:** 10.1371/journal.pone.0225397

**Published:** 2020-01-07

**Authors:** Muhammad Tayyib, Muhammad Amir, Umer Javed, M. Waseem Akram, Mussyab Yousufi, Ijaz M. Qureshi, Suheel Abdullah, Hayat Ullah

**Affiliations:** 1 Faculty of Engineering and Technology, International Islamic University Islamabad, Islamabad, Pakistan; 2 Institute of Fundamental and Frontier Science, University of Electronic Science and Technology of China, Chengdu, China; 3 Department of Electrical Engineering, Air University, Islamabad, Pakistan; Ulm University, GERMANY

## Abstract

Wearable electronics capable of recording and transmitting biosignals can provide convenient and pervasive health monitoring. A typical EEG recording produces large amount of data. Conventional compression methods cannot compress date below Nyquist rate, thus resulting in large amount of data even after compression. This needs large storage and hence long transmission time. Compressed sensing has proposed solution to this problem and given a way to compress data below Nyquist rate. In this paper, double temporal sparsity based reconstruction algorithm has been applied for the recovery of compressively sampled EEG data. The results are further improved by modifying the double temporal sparsity based reconstruction algorithm using schattern-p norm along with decorrelation transformation of EEG data before processing. The proposed modified double temporal sparsity based reconstruction algorithm out-perform block sparse bayesian learning and Rackness based compressed sensing algorithms in terms of SNDR and NMSE. Simulation results further show that the proposed algorithm has better convergence rate and less execution time.

## Introduction

In Brain Computer Interface (BCI), a non-muscular connection between computers and human is made to assist in conversion of coded brain signals into external commands [25, 32]. EEG based BCI has shown significant importance in recent years for health-care monitoring, including early detection of seizure, trauma, alzheimer and stroke [29]. Normal EEG signal contain large number of data that cannot be sampled and transmitted during many real life scenarios. Epileptic seizure detection of patients require continuous EEG monitoring that may last upto a number of hours [22]. Saving, processing and transmission of this huge data requires bulk storage and immense processing power [1]. As an example, multichannel EEG recording ranges from 24 to several hundred electrodes. With 24 electrodes, if sampled at 200 HZ using 12 bits of resolution, it produces a data of at least 1 GB per day [8]. Compressive sensing (CS) theory suggests solution to this problem by taking samples far fewer than the Nyquist rate along with faithful recovery [38].

Traditional way of compression discards huge amount of data resulting in a lossy compression. To overcome this issue, signal compression techniques with better sampling patterns have been developed, which enables to store the same amount of data in more compact form [24]. Some methods are matched filters, autocorrelation based euclidian distance, bayesian interface methods, wavelet compression and jpeg 2000 [4]. All these methods need sampling measurements at Nyquist rate using analog to digital converter (ADC), resulting in computationally complex data, high processing time, and expensive hardware. In order to mitigate all these problems, CS provides a promising solution [4]. CS reconstructs from highly under-sampled data, even below the Nyquist rate, discarding the redundant information. This results in a huge reduction in dimension due to less number of measurements. The basic theory of CS rely on two necessary conditions, sparsity and incoherence [14].

The main idea behind CS is that, signal can be represented by only few non-zero coefficients, this is done by using some sparse sensing matrix [11]. For CS, two assumptions are made. Firstly, either data is itself sparse or sparse in some transform domain. Secondly, the measurement basis and representation basis are mutually incoherent [9], this results in a compression below the Nyquist rate. As number of measurements are far fewer than original signal, thus recovering the original signal is an NP-hard problem [23]. Due to non-sparse representation of EEG in time domain, EEG signal is made sparse by using different basis or dictionary functions. There have been many publications indicating dictionaries such as slepian basis and Gabor framework [38]. Hesham [19], presented the CS framework for EEG using dirac sensing matrix, and efficiently reconstructed the EEG signal after compression. Angshul [10], illustrated CS recovery of EEG using 2-D fourier transform, however [38], claimed that better reconstruction can be achieved using wavelet domain instead of Gabor domain. Jun [3], claimed that using daubechies wavelets, the reconstruction accuracy achieved is better than that of other basis functions.

### Compressed sensing

Compressed sensing depend on the hypothesis that the signal **x** is compressed by $\Phi \in R^{M\times N}$ (sampling or measurement matrix). The sampling model is formulated as,

$$\begin{array}{c} y=\Phi x+\xi \end{array}$$ (1)

where $y\in R^{M\times 1}$ represent the compressed measurements with M≪N, indicating that number of sampled measurements are far less than the original signal. If **x** is sparse, then recovery problem only requires the compressed measurements and sampling matrix, but if not, than signal **x** should be sparse (transformed) in representation matrix (dictionary) $\Psi \in R^{N\times P}$ with N≪P. This can be written as,

$$\begin{array}{c} x=\Psi \theta \end{array}$$ (2)

where $\theta \in R^{P\times 1}$ is sparse. **x** can be recovered using measurements **y**, dictionary **Ψ**, and sampling matrix **Φ**. The minimization problem formed is,

$$\begin{array}{c} \begin{array}{cc} \text{min}\;{\left\|\theta \right\|}_{0} & \;\;\;s.t\;\;\;y=\Phi \Psi \theta \end{array} \end{array}$$ (3)

where ‖.‖_0_ is the *ℓ*_0_ norm, i.e it counts the number of non-zero entries. The **x** is called K-sparse when number of non-zero entries are vector equal to K. The task of recovering **x** from measurements **y** is an under-determined inverse problem, and finding its solution is NP- hard problem [37], as the sensing matrix **Ψ** due to large undersampling is ill conditioned. So general problem is regularized in order to achieve recovery. Sparsity regularization is comparatively elementary solution to this problem.

In sparse recovery problem, the expected signal is said to be sparse by representing it with transform **Ψ**, to regularize it in transformed domain, *ℓ*_1_ norm is used as the substitute for *ℓ*_0_ norm. Hence, for EEG reconstruction problem, the sparsity regularization is formulated as [21],

$$\begin{array}{c} \hat{x}=arg\;\underset{x}{\text{min}}\;{\left\|y-\Phi \Psi \theta \right\|}_{F}^{2}+\lambda_{1}{\left\|\theta \right\|}_{1} \end{array}$$ (4)

**Φ** represent the basis of sparsifying transform and ‖.‖_1_ is *ℓ*_1_ norm. The minimization of variance of noise is done with fedility term in Eq 4. The regularization term λ_1_ is added to induce sparsity on **x** over basis **Ψ**, and ${\left\|.\right\|}_{F}^{2}$ represents the Frobenius norm, which can be defined as,

$$\begin{array}{c} {\left\|\theta \right\|}_{F}^{2}=Tr\left[\left(\theta \right){\left(\theta \right)}^{T}\right] \end{array}$$ (5)

**Fig 1:**
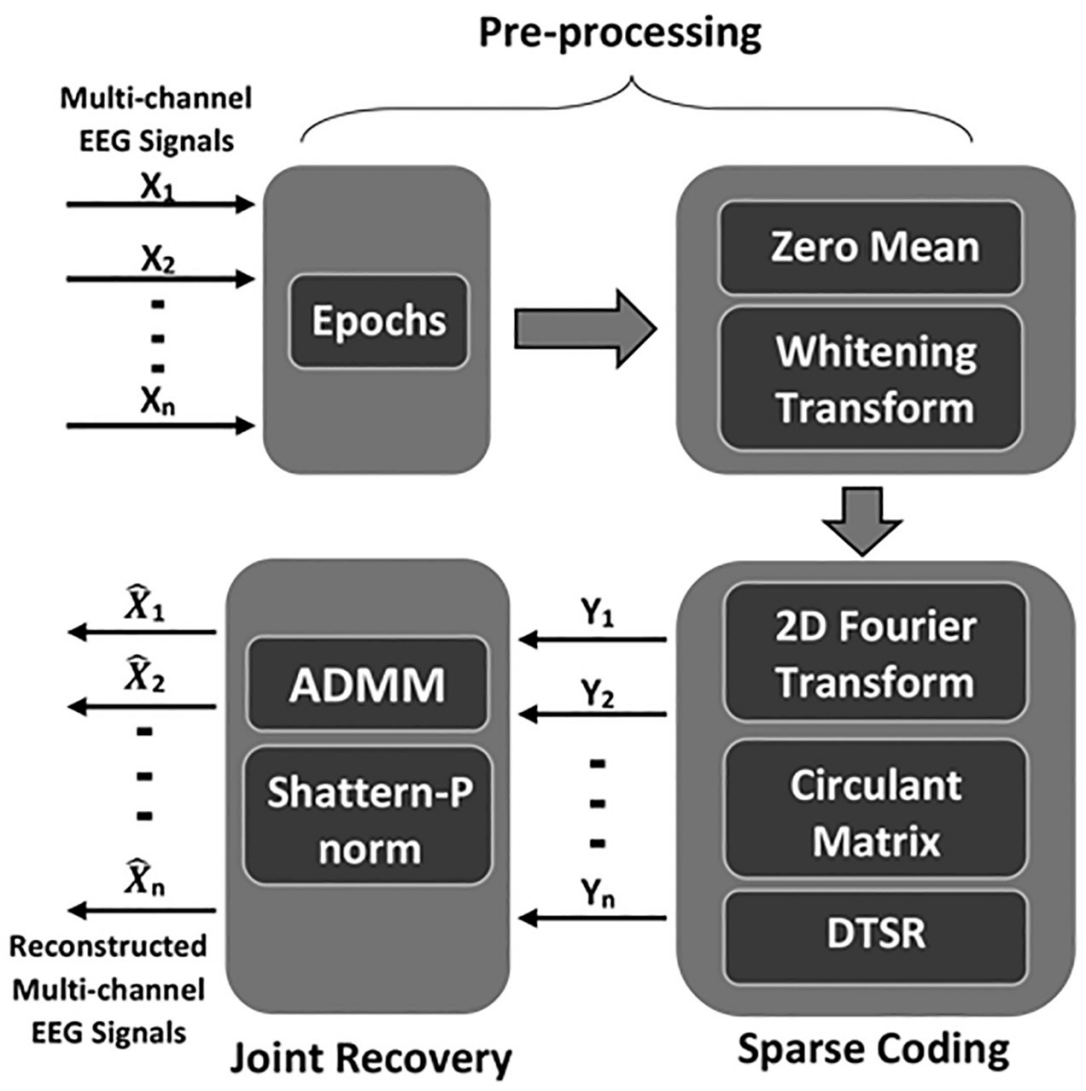
Flow diagram of proposed method.

The proposed method is based on double sparsity based framework. The motivation behind proposed method is to increase the existing reconstruction accuracy of multi-channel EEG signals using following contributions: First, the multi-channel EEG signal is pre-processed using zero mean and whitening transform. The total variation matrix exploited in previous work exhibits redundancy in reconstruction accuracy, so instead of using total variation matrix, proposed algorithm explored the concept of circulant matrix as a sparse sensing matrix. In addition, the shattern-p norm is used as non-convex surrogate function to exploit the double sparsity in CS recovery of multi-channel EEG signals. The flow diagram of proposed method is shown in Fig 1.

The rest of the paper is organized as follows, section III summarize the existing methods used for the reconstruction of compressively sampled EEG signals. Section IV includes the discussion and analysis of proposed method on the basis of quantitative analysis, section V gives the concluding remarks.

## Related work

### Rackness based compressive sensing

Power consumption during wireless transmission has been focus of many researchers, Nicola et al. [16] worked on solving the power consumption issue by introducing the rackness based compressed sensing. Using rackness based CS, good signal reconstruction of EEG signal can be achieved. Rackness approach is based on assumption that certain signal exhibits non-flat energy distribution. Using this assumption, there is no need to construct **Φ** from randomly selected i.i.d entries. Instead of constructing randomly, **Φ** is tuned statistically which match with the input signal. This property increases the average energy of **y**, which ultimately increases the reconstruction accuracy. To formulate this property, rackness *ρ* between two stochastic process **x** and **Φ** is defined as,

$$\begin{array}{c} \rho \left(\Phi ,x\right)=E_{\left(\Phi ,x\right)}\left[\right|\left\langle \Phi_{j},x\right\rangle \left|\right] \end{array}$$ (6)

where the static expectation over **x** and **Φ** is represented by *E*_(**Φ**,**x**)_, with 〈.〉 as standard inner product, ****Φ**_*j*_** is the sensing sequence, and **x** is the signal instances. Using rackness based approach, both noise suppression and good signal reconstruction is achieved.

### Block sparse bayesian learning

Using field programmable gate array (FPGA), Liu et al. [27] proved that comparing to wavelet domain, block sparse bayesian learing (BSBL) shows prominent results in terms of power consumption and reconstruction accuracy. Using fast marginalized (FM) likelihood method, fast implementation of BSBL was developed in which EEG signal is structured into blocks as shown in Eq (7),

$$\begin{array}{c} x=[\underset{x_{1}^{T}}{\underset{︸}{x_{1},\ldots .,x_{d_{1}}}},\ldots ‥,\underset{x_{g}^{T}}{\underset{︸}{x_{1},\ldots .,x_{d_{g}}}}] \end{array}$$ (7)

which represents that **x** has g blocks, with few non-zero blocks and *d*_*i*_ is the size of ith block. The BSBL uses intra-block structure correlation to model the signal **x** with Gaussian distribution. The resulting reconstruction is not robust to all methods, as blocking introduces noise, therefore, regularization is done in order to achieve better results.

### Simultaneous co-sparsity and low rank

In a very recent work [2], it was discussed that due to correlation in EEG signals, same sparsity pattern is adopted after transformation i.e. the values of transform coefficients have values at same positions leading to row-sparse recovery [2]. This theory was formulated using *ℓ*_2,1_ norm minimization problem.

$$\begin{array}{c} \begin{array}{cc} \hat{X}=arg\;\underset{X}{\text{min}} & \|y-\Phi F^{T}{X\|}_{F}^{2}+\lambda {\left\|X\right\|}_{2,1} \end{array} \end{array}$$ (8)

where *ℓ*_2,1_ norm is the sum of the rows of *ℓ*_2_ norm. In Eq 8, *ℓ*_2_ norm gives the dense solution in selected rows, hence sum of *ℓ*_2_ norm promotes selection of very few rows.

### Blind compressed sensing

The theory of CS relies on the assumption that sparsity of the signal is known in some basis. The Blind Compress Sensing (BCS) [17], avoid this assumption by using both CS and dictionary learning. BCS estimates the sparse signal as well as the sparsifying dictionary from the data. The assumption that data is sparse in learned dictionary, i.e. **X** = **D**J where J are the sparse coefficients and **D** is the unknown dictionary (to be estimated) [17].

$$\begin{array}{c} \begin{array}{cc} \hat{X}=\underset{D,J}{\text{min}} & {\left\|y-\text{AD}J\right\|}_{F}^{2}+\lambda_{1}{\left\|D\right\|}_{F}^{2}+\lambda_{2}{\left\|vec\left(J\right)\right\|}_{1} \end{array} \end{array}$$ (9)

In BCS [17, 35], both signal estimation and dictionary proceeds simultaneously.

### Double temporal sparsity based reconstruction

Using double temporal sparsity reconstruction (DTSR), better reconstruction can be achieved with acceleration in time. Priya [28] proposed DTSR for sparse signal recovery of fMRI data with prominent results. In this work we have sued a modified form of DTSR along with some pre-processing for sparse recovery of EEG signal.

The DTSR algorithm uses total variation based algorithm and imposes two *ℓ*_1_ norm constraints. First constraint is applied on transformed domain of temporal data and other is imposed on the consecutive difference of same data. The cost function of Eq 4 can be written as,

$$\begin{array}{c} \begin{array}{cc} \hat{x}=arg & \underset{x}{\text{min}}\;{\left\|y-\Phi F\theta \right\|}_{F}^{2}+\lambda_{1}{\left\|\Psi x\right\|}_{1}\\ & +\lambda_{2}\sum_{t=2}^{T}\left|x_{t}-x_{t-1}\right| \end{array} \end{array}$$ (10)

where λ_1_, λ_2_ are positive regularization terms, F is the 2-D Fourier transform while the third term shows the consecutive difference of columns of data. The matrix formulation of a Eq 10 can be shown as

$$\begin{array}{c} \begin{array}{cc} \hat{x}=arg & \underset{x}{\text{min}}\;{\left\|y-\Phi F\theta \right\|}_{F}^{2}+\lambda_{1}{\left\|\Psi x\right\|}_{1}\\ & +\lambda_{2}{\left\|\text{xD}\right\|}_{1} \end{array} \end{array}$$ (11)

where **D** shows the consecutive difference on the successive columns of **x**, known as total variation temporal sparsity shown as

$$\begin{array}{c} D=[\begin{array}{ccccccc} -1 & 1 & 0 & . & . & 0 & 0\\ 0 & -1 & 1 & 0 & . & . & 0\\ 0 & 0 & -1 & 1 & . & . & .\\ 0 & 0 & . & . & . & . & .\\ . & . & . & . & . & . & 0\\ 0 & . & . & . & 0 & -1 & 1\\ 0 & 0 & . & 0 & 0 & 0 & -1 \end{array}] \end{array}$$

## Proposed method

**Fig 2:**
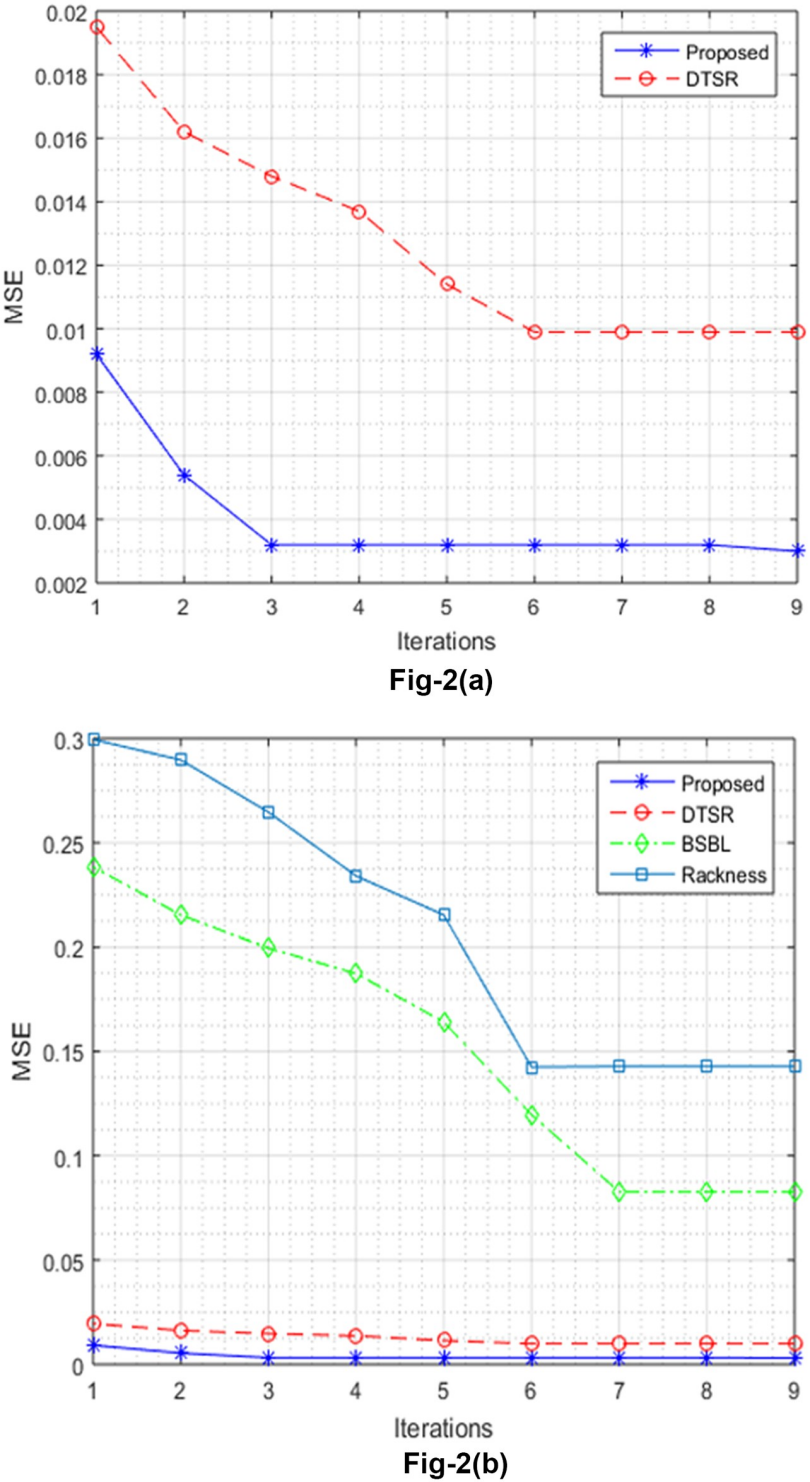
MSE vs iterations. A: Original vs proposed method. B: Comparison with related methods.

In this section, alternating direction multiplier method (ADMM) is modified on the basis of desired EEG signal recovery problem using DTSR. In the pre-processing of signal **x**, first of all signal is made zero mean and then it is made white by making each column of signal un-correlated to each other. The unit variance and zero mean can be expressed in mathematical terms as,

$$\begin{array}{c} M=\frac{x-\mu }{\sigma } \end{array}$$ (12)

where *μ* is the mean, and *σ* is the variance of EEG signal **x**, this is done in fashion that each value of **x** is subtracted form mean of individual columns of **x**, and divided by *σ* resulting EEG signal with zero mean. This zero mean EEG data is made white by using optimal whitening method [30]. The reason behind this step is that close channel in multichannel EEG signal exhibits strong correlation, removing this correlation by making the columns of EEG signal orthogonal to each other results in less search time in optimal sparse signal recovery. This can be shown in Fig 2, where zero-mean and white data becomes stable in less number of iteration than original algorithm. The whitening of zero-mean signal can be shown mathematically as

$$\begin{array}{c} Z={\left(z_{1},\ldots ,z_{d}\right)}^{T}=WM \end{array}$$ (13)

where d is the dimension of zero-mean vector, W is the d×d whitening matrix. Whitening in general terms can be viewed as [30],

$$\begin{array}{c} Z=V^{-1/2}M \end{array}$$ (14)

where V is the variance, $\text{V}=\text{diag}(\sigma_{1}^{2},\ldots ,\sigma_{d}^{2})$, such that, $\text{var}(m_{i})=\sigma_{i}^{2}$. The whitening transform should follow, WΣ*W*^*T*^ = I and thus W(Σ*W*^*T*^W) = W, which is only achieved if W satisfy the constraint

$$\begin{array}{c} W^{T}W=\Sigma^{-1} \end{array}$$ (15)

Using Mahalanobis whitening method [30], the whitening transform employed in this work is

$$\begin{array}{c} W^{ZCA}=\Sigma^{-1/2} \end{array}$$ (16)

The difference between zero-mean and whitened data can be seen by number of iterations in Fig 2. Instead of using total variation matrix **D** used in Eq 11, in this paper we explored the idea of using circulant matrix [13]. For CS, the binary measurement matrices are formed using parity check matrix of array coding. A parity matrix **H**(r,q) is the identity matrix of dimension r×q and (i,j)-th circulant permutation matrix **P**^(*i*−1)(*j*−1)^ with q as odd prime and r as positive integer, such that 1≤r≤q [36]. The i-th row circulant matrix is formed by cyclic shift of (*i*+1)-th or (*i*-1)-th row by one term. This can be shown as,

$$\begin{array}{c} H\left(r,q\right)=[\begin{array}{ccccccc} I & I & & . & . & I\\ I & P & & . & . & P^{q-1}\\ . & . & & . & & .\\ . & . & & & . & .\\ . & . & & & & . & \\ I & P^{r-1} & & . & . & P^{\left(r-1)(q-1\right)} \end{array}] \end{array}$$

$$\begin{array}{c} P=[\begin{array}{ccccccc} 0 & 1 & 0 & . & . & . & 0\\ 0 & 0 & 1 & . & . & . & 0\\ . & . & . & . & & & .\\ . & . & . & & . & & .\\ . & . & . & & & . & .\\ 0 & 0 & 0 & . & . & . & 1\\ 1 & 0 & 0 & . & . & . & 0 \end{array}] \end{array}$$

After making these changes in the cost function of DTSR, the modified version of algorithm can be rewritten as,

$$\begin{array}{c} \begin{array}{cc} \hat{z}=arg\;\underset{z}{\text{min}} & {\left\|y-\Phi Fz\right\|}_{F}^{2}+\lambda_{1}{\left\|\Psi z\right\|}_{1}\\ & +\lambda_{2}{\left\|\text{zH}\right\|}_{1} \end{array} \end{array}$$ (17)

Instead of using frobenious norm, which is basically square root of the sum of the absolute squares defined in Eq 11, we have used the schattern-*p* norm. Schattern-*p* norm has been successfully used for sparse synthesis model and shows accurate results [3, 7, 18, 33]. Eq 17 can be re-written as

$$\begin{array}{c} \begin{array}{cc} \hat{z}=arg\;\underset{z}{\text{min}} & {\left\|y-\Phi Fz\right\|}_{S_{p}}^{p}+\lambda_{1}{\left\|\Psi z\right\|}_{1}\\ & +\lambda_{2}{\left\|\text{zH}\right\|}_{1} \end{array} \end{array}$$ (18)

where ${\left\|.\right\|}_{S_{p}}^{p}$ is the schattern-*p* norm and it is the sum of all singular values *σ* of data **z** upto value *p*, for matrix $T\in H_{m}$ and p∈[1,+∞], ${\left\|.\right\|}_{S_{p}}^{p}$ is defined as,

$$\begin{array}{c} {\left\|T\right\|}_{{}_{p}}≔{\left(|\sum_{i}\sigma_{i}{\left(T\right)|}^{p}\right)}^{1/p} \end{array}$$ (19)

where $H_{m}$ is monotone Hilbert space, and *σ* is the singular values of **T** in non-increasing order such that *σ*_1_(**T**) ≥ *σ*_2_(**T**)….

To solve Eq 18, optimized solution is obtained using ADMM [6].

### Optimization algorithm

Strained optimization problem in recent literature [20, 26, 34]. The ADMM ease the solution by breaking down the original cost function into several objective function, that are comparatively easy to solve.

Following [28], two auxiliary matrices $P\in R^{M\times N}$ and $Q\in R^{M\times N}$ are introduced in Eq 18 as

$$\begin{array}{c} \begin{array}{cc} \hat{z}=arg\;\underset{z}{\text{min}} & {\left\|y-\Phi Fz\right\|}_{S_{p}}^{p}+\lambda_{1}{\left\|P\right\|}_{1}+\lambda_{2}{\left\|Q\right\|}_{1}\\ & s.t\;\;\;\;\;P=\Psi z,\;Q=\text{zH} \end{array} \end{array}$$ (20)

By adding these new constraints for each of auxiliary matrices, the objective function formed is

$$\begin{array}{c} \begin{array}{cc} \hat{z} & =arg\;\underset{z}{\text{min}}\;{\left\|y-\Phi Fz\right\|}_{S_{p}}^{p}+\lambda_{1}{\left\|P\right\|}_{1}+\lambda_{2}{\left\|Q\right\|}_{1}\\ & +\frac{\eta_{1}}{2}\|P-\Psi z-B_{1}{\|}_{F}^{2}+\frac{\eta_{2}}{2}{\left\|Q-\text{zH}-B_{2}\right\|}_{F}^{2} \end{array} \end{array}$$ (21)

where B_1_, B_2_ are lagrange multipliers to satisfy the equality auxiliary and original matrices, and *η*_1_, *η*_1_ are penalty parameters.

ADMM updates variables **P**, **Q** and **z** alternatively in the above lagrange function. By keeping the other two variables fixed, one variable is minimized in each iteration. Thus, the above function can be decomposed into three sub-problems, with new objective function as,

$$\begin{array}{c} A_{1}\;\;:\;\;\;\;arg\;\underset{P}{\text{min}}\lambda_{1}{\left\|P\right\|}_{1}+\frac{\eta_{1}}{2}{\left\|P-\Psi z^{j-1}-B_{1}^{j-1}\right\|}_{F}^{2} \end{array}$$ (22)

$$\begin{array}{c} A_{2}\;\;:\;\;\;\;arg\;\underset{Q}{\text{min}}\lambda_{2}{\left\|Q\right\|}_{1}+\frac{\eta_{2}}{2}{\left\|Q-z^{j-1}H-B_{2}^{j-1}\right\|}_{F}^{2} \end{array}$$ (23)

$$\begin{array}{c} \begin{array}{cc} A_{3}\;\;:\;\;\;\;arg\;\underset{z}{\text{min}} & {\left\|Y-\Phi Fz\right\|}_{S_{p}}^{p}+\frac{\eta_{1}}{2}{\left\|P^{j}-\Psi z-B_{1}^{j-1}\right\|}_{F}^{2}\\ & +\frac{\eta_{2}}{2}{\left\|Q^{j}-\text{zH}-B_{2}^{j-1}\right\|}_{F}^{2} \end{array} \end{array}$$ (24)

where j is the number of iterations, *A*_1_ and *A*_2_ subproblems minimize objective function over **P** and **Q** respectively with fixed **z**. Similarly, *A*_3_ minimizes **z** keeping **P** and **Q** fixed. Subproblems *A*_1_, *A*_2_ and *A*_3_ are solved iteratively by updating the lagrange multipliers B_1_ and B_2_.

#### *A*_1_ and *A*_2_ subproblems

Subproblems *A*_1_ and *A*_2_ are *ℓ*_1_ minimization problem, for general *ℓ*_1_ minimization problem as

$$\begin{array}{c} \underset{U}{\text{min}}\;{\alpha \|U\|}_{1}+\frac{\beta }{2}{\left\|U-V\right\|}_{F}^{2} \end{array}$$ (25)

the solution is [28],

$$\begin{array}{c} U=Soft(V,2\frac{\alpha }{\beta }W) \end{array}$$ (26)

where **W** is unitary matrix and $U,V\in R^{M\times N}$ with *α*, *β* > 0. **V** in Eq 25 is initial approximate of **U**. Hence for j iteration, the solution for subproblem *A*_1_ is

$$\begin{array}{c} P^{j}=Soft\left(\left(\Psi z^{j-1}+B_{1}^{j-1}\right),2\frac{\lambda_{1}}{\eta_{1}}W\right) \end{array}$$ (27)

For j iteration **P** is computed for subproblem *A*_1_. Similarly **Q** is computed for subproblem *A*_2_ using **z**^*j*−1^ and ${\text{B}}_{2}^{j-1}$ as

$$\begin{array}{c} Q^{j}=Soft\left(\left(z^{j-1}H+B_{2}^{j-1}\right),2\frac{\lambda_{2}}{\eta_{2}}W\right) \end{array}$$ (28)

#### *A*_3_ subproblem

Subproblem *A*_3_ is quadratic, to solve it conjugate gradient algorithm is used [12, 15]. In this paper, line search conjugate gradient algorithm is used [28]. This algorithm is iterative in which descent direction is selected on the minimization of function. Further, step size is determined by using line search method. For general quadratic equations, line search conjugate gradient algorithm gives the finite convergence.

#### Updating lagrange multiplier

In the last step lagrange multiplier is updated iteratively. Lagrange multiplier helps in achieving the convergence in subsequent iteration. The pseudo algorithm of proposed method is shown in Algorithm 1. The convergence is achieved by comparing convergence of objective function with threshold or with maximum number of iterations achieved.

**Algorithm 1** Proposed Algorithm

1) **INPUT**: λ_1_, λ_2_, ${\text{B}}_{1}^{0}$, ${\text{B}}_{2}^{0}$, Z^0^, j = 1

2) **while** Convergence do not met **do**

3) Solve P for subproblem *A*_1_ using 27

4) Solve Q for subproblem *A*_2_ using 28

5) Solve Z for subproblem *A*_3_ using 24

6) Updating lagrange multipliers

${\text{B}}_{1}^{j}={\text{B}}_{1}^{j-1}+\psi {\text{X}}^{j}-{\text{P}}^{j}$

${\text{B}}_{2}^{j}={\text{B}}_{2}^{j-1}+{\text{X}}^{j}\;\text{H}-{\text{Q}}^{j}$

7) j = j+1

8) **end while**

8) **OUTPUT**: Reconstructed signal $\hat{Z}$

## Results and discussion

### EEG dataset

The publicly available EEG dataset [5] is used for the purpose of sparse signal recovery of multi-channel EEG signal. This commonly used EEG dataset contain 32 channel EEG signal of length 30720 data points, with each signal of channel contain 80 epochs and 384 points. For compression of each epoch, sensing matrix $\Phi \in R^{192\times 384}$ is used as sparse circulant matrix and $\Psi \in R^{384\times 384}$ is used as Fourier domain sparsifying matrix formed by calculating the Fourier transform of **z** along each row. To recover EEG signal, ADMM algorithm is used [6].

### Quantitative analysis

This section includes the results of the proposed modified DTSR method in comparison with few of the existing CS-based EEG signal reconstruction techniques. For the purpose of reconstruction quality measurement, normalized mean square error (NMSE), mean square error (MSE) and signal to noise distortion ratio (SNDR) are used.

For reference EEG signal **x** and its reconstructed version $\hat{x}$, the NMSE is computed as

$$\begin{array}{c} NMSE=\frac{\|x-\hat{x}{\|}_{2}^{2}}{{\left\|x\right\|}_{2}} \end{array}$$ (29)

where ‖.‖_2_ represent the *ℓ*^2^-norm. Similarly SNDR is calculated as

$$\begin{array}{c} SNDR=10\log_{10}\frac{\|\hat{x}{\|}_{2}^{2}}{\|\hat{x}{-x\|}_{2}^{2}} \end{array}$$ (30)

MSE can be calculated as

$$\begin{array}{c} MSE=\sum_{l=1}^{L}\frac{\|\hat{x}{-x\|}_{F}^{2}}{LNC} \end{array}$$ (31)

where **x** and $\hat{x}$ are the reference and reconstructed EEG signals, C is the number of EEG channels, N is the length of epoch, and *L* is the number of experiments.

**Fig 3:**
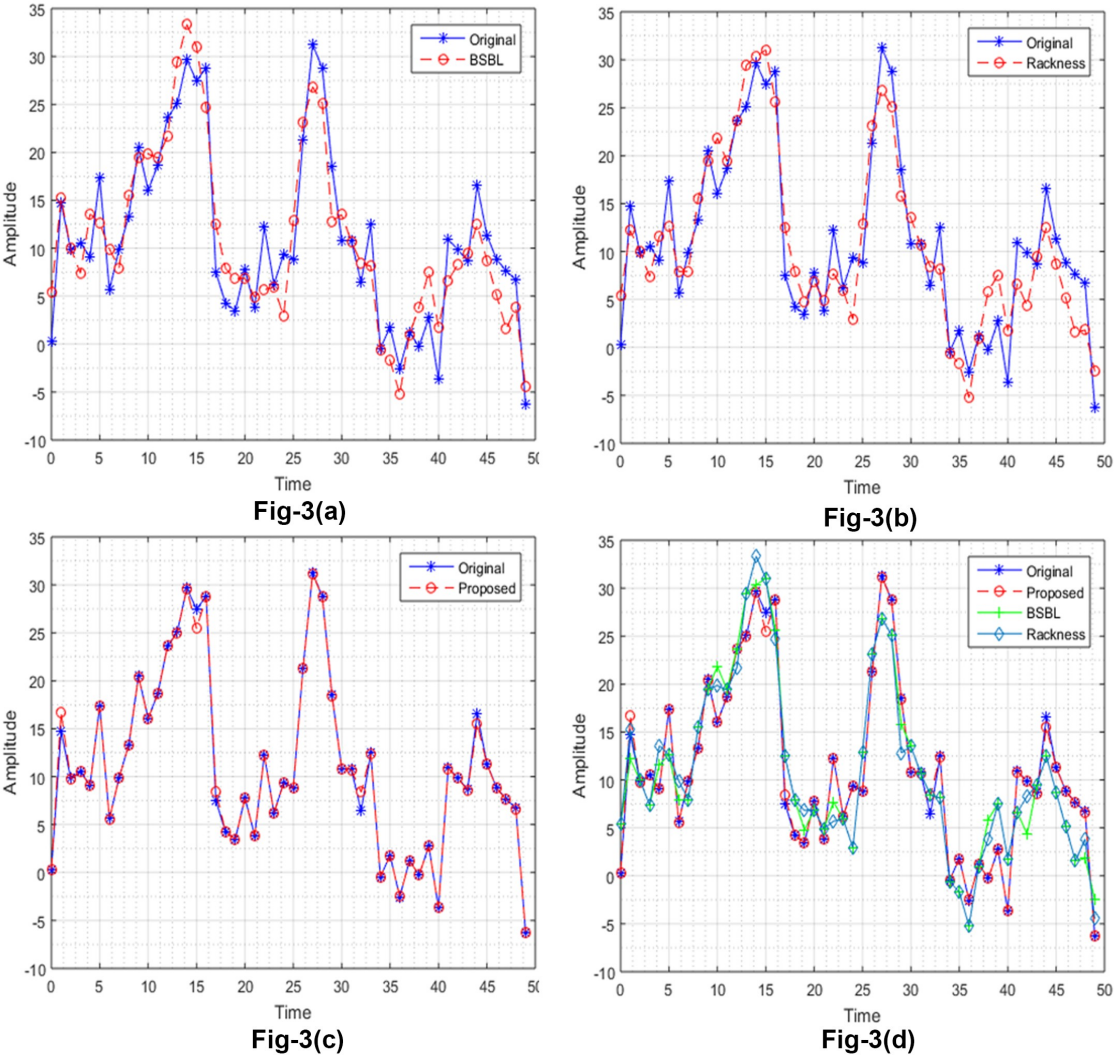
Reconstruction. Overlayed original and reconstructed EEG signal for duration 0-50secs A: BSBL. B: Rackness. C:Proposed D:Combined.

Reconstructed NMSE (average), MSE (average), band SNDR (average) for all 32 channel EEG signal is presented in Table 1. The compression rate of 25%(4:1), and 50%(2:1) is used for evaluation. The MSE of multi-channel EEG signal is shown in Fig 2. The proposed method gives best results in less number of iteration than other existing algorithms. Multi channel EEG signal along with its reconstructed versions with different algorithms are shown in Fig 3. EEG multi channel data used in this analysis consists of 32 channels, 384 time series and 80 epochs represented by 32×384×80. The results shown in Fig 3 and Table 1 indicates that the proposed algorithm outperforms in terms of accuracy and execution time as compared to other state of the art algorithms.

**Table 1:** Quantitative measures for sparse signal recovery of EEG signals.

Authors	CR	MSE	NMSE	SNDR(db)
Zhilin et al. [25]	2:1	0.1452	0.118±0.047	16.23
	4:1	0.2804	0.89±0.05	17.86
Fabio et al. [31]	2:1	0.1960	0.116±0.046	16.45
	4:1	0.3920	0.85±0.08	18.29
Priya et al. [28]	2:1	0.0108	0.076±0.005	23.62
	4:1	0.0216	0.157±0.015	19.25
**Proposed**	2:1	**0.0024**	**0.053±0.011**	**24.63**
	4:1	**0.0063**	**0.105±0.014**	**21.76**

## Conclusion

In this work compressively sampled EEG data is recovered using DTSR algorithm. Conventional DTSR algorithm which was originally designed for fMRI data is tailored for application to EEG sparse recovery by making three main contributions. As a first step pre-processing is done by making the EEG data zero mean and unit variance. Second step is to formulate circulant matrix instead of total variation matrix for limiting the search space for fast convergence of the algorithm. Finally it is shown that instead of frobenius norm, using shattern-p norm yields better reconstruction accuracy. The proposed modified DTSR algorithm outperforms conventional DTSR as well as other state of the art CS recovery techniques in terms of NMSE and SNDR.
